# Design, implementation and initial findings of COVID-19 research in the Rotterdam Study: leveraging existing infrastructure for population-based investigations on an emerging disease

**DOI:** 10.1007/s10654-021-00789-7

**Published:** 2021-07-17

**Authors:** Silvan Licher, Natalie Terzikhan, Marije J. Splinter, Premysl Velek, Frank J. A. van Rooij, Jolande Verkroost-van Heemst, Annechien E. G. Haarman, Eric F. Thee, Sven Geurts, Michelle M. J. Mens, Niels van der Schaft, Maud de Feijter, Luba M. Pardo, Brenda C. T. Kieboom, M. Arfan Ikram

**Affiliations:** 1 Department of Epidemiology, Erasmus MC - University Medical Center Rotterdam, Rotterdam, The Netherlands; 2 Department of Ophthalmology, Erasmus MC - University Medical Center Rotterdam, Rotterdam, The Netherlands; 3 Department of Dermatology, Erasmus MC - University Medical Center Rotterdam, Rotterdam, The Netherlands; 4 Department of General Practice, Erasmus MC - University Medical Center Rotterdam, Rotterdam, The Netherlands

**Keywords:** COVID-19, Population-based, Risk factors, Prevalence, Design, Methods

## Abstract

The Rotterdam Study is an ongoing prospective, population-based cohort study that started in 1989 in the city of Rotterdam, the Netherlands. The study aims to unravel etiology, preclinical course, natural history and potential targets for intervention for chronic diseases in mid-life and late-life. It focuses on cardiovascular, endocrine, hepatic, neurological, ophthalmic, psychiatric, dermatological, otolaryngological, locomotor, and respiratory diseases. In response to the COVID-19 pandemic, a substudy was designed and embedded within the Rotterdam Study. On the 20th of April, 2020, all living non-institutionalized participants of the Rotterdam Study (*n* = 8732) were invited to participate in this sub-study by filling out a series of questionnaires administered over a period of 8 months. These questionnaires included questions on COVID-19 related symptoms and risk factors, characterization of lifestyle and mental health changes, and determination of health care seeking and health care avoiding behavior during the pandemic. As of May 2021, the questionnaire had been sent out repeatedly for a total of six times with an overall response rate of 76%. This article provides an overview of the rationale, design, and implementation of this sub-study nested within the Rotterdam Study. Finally, initial results on participant characteristics and prevalence of COVID-19 in this community-dwelling population are shown.

**Supplementary Information:**

The online version contains supplementary material available at 10.1007/s10654-021-00789-7.

## Introduction

The ongoing COVID-19 pandemic has had huge impact on society, both in terms of morbidity and mortality due to COVID-19, as on physical and psychosocial health. As the disease spread across populations from late 2019 onwards, so did a multitude of research activities aimed at better understanding of all aspects of SARS-CoV-2 and the associated COVID-19 disease. Preclinical studies aimed to unravel etiologic mechanisms and diagnostic biomarkers, clinic-based studies among COVID-19 patients targeted clinical course and management, and trials were set-up to identify effective preventive or therapeutic interventions [1].

Against this backdrop of expanding knowledge about this emerging disease, several areas remain understudied. First, it remains unclear which host-determinants, such as genomics, microbiome, metabolome and lifestyle aspects, are important for contracting SARS-CoV-2 and determining the (sub)clinical manifestation of COVID-19. Second, long-term effects of COVID-19, especially in conjunction with co-morbidity and polypharmacy among older adults, are unknown. Third, long-term effects of governmental countermeasures against the spread of SARS-CoV-2 are expected, but have been quantified limitedly on a population-level. This involves consequences on non-COVID-19 related physical health, such as delays in healthcare seeking behaviour, as well as the impact on psychosocial health, such as isolation [2, 3]. Cohort studies embedded within the general community that continuously follow study participants over a long duration are ideally suited to address these knowledge gaps.

The Rotterdam Study is an ongoing population-based cohort study with rich phenotyping, appropriate representation of the underlying source population [4, 5], and a robust existing infrastructure [6]. Soon after the spread of SARS-CoV-2 in the Netherlands, the Rotterdam Study set up a sub-study based on repeated questionnaires and aimed at addressing various aspects of COVID-19, including related symptoms and risk factors, lifestyle and mental health and health care seeking behaviour and utilization during the pandemic. This article details the design, considerations, and implementation of the COVID-19 sub-study within the Rotterdam Study. We also showcase initial results on characteristics of the study participants and prevalence of COVID-19 in this community.

## Methods

### The Rotterdam study

The Rotterdam Study is a prospective cohort study that started in 1989 among residents of Ommoord, a well-defined district in the city of Rotterdam, the Netherlands [6]. The initial cohort comprised of 7983 persons who were 55 years of age and over (response rate: 78% of 10,215 invitees). There were no pre-specified exclusion criteria and all persons older than 55 years of age living in the area were invited to participate (‘RS I’). In 2000, the cohort was extended with residents of Ommoord who had become 55 years of age and over and who had not been invited before (‘RS II’). A total of 3011 of men and women were included (response rate 67% of 4472). In 2006, the cohort was extended with a third wave (‘RS III’), including 3932 persons aged 45 years and over (response rate 65% of 6057). A further extension of the cohort was initiated in 2016 (‘RS IV’), in which 3,368 residents of Ommoord aged 40 years and over were included (response rate 46%). The participants were all extensively examined at baseline and at subsequent follow-up visits that took place every three to six years, as described previously.(6) On April 8th 2020, 9008 out of a total of 18,924 enrolled participants (47.6%), were still alive and participating within the Rotterdam Study. Of those, 8,732 participants (96.9%) were not institutionalized or living in nursing homes, and thus invited for participation on April 20th 2020, in the current sub-study (Supplementary Information, eFig. 1). Results have been reported in accordance with STROBE guidelines.

**Fig. 1:**
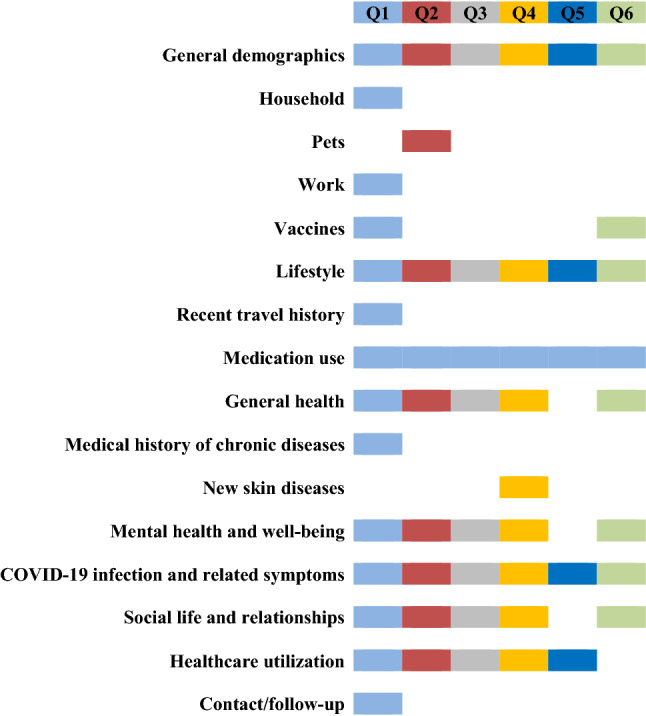
Overview of the different inquired questionnaire domains over time, segregated by each COVID-19 questionnaire

### The sub-study

This sub-study is embedded in the Rotterdam Study, and consisted of a series of questionnaires to cover the following categories: COVID-19 related symptoms and risk factors; lifestyle and mental health; and health care seeking behavior and utilization during the pandemic (see Fig. 1 for an overview of questionnaire domains, with detailed descriptions of each domain; the complete datadictionary of the questionnaire is provided in the Supplementary Information).

As of May 2020, the questionnaires have been sent out repeatedly for a total of six times. The length of the intervals between the consecutive questionnaires was based on the actual infection curve in the Netherlands, with initially 2-week intervals in April 2020 (following the first peak of COVID-19 in the Netherlands), which has been extended to 4-week intervals by mid-May 2020, up to 8-week intervals from August to October 2020. The first two questionnaires (‘Q1’ and ‘Q2’) were sent on paper to all participants, all participants were asked if they want to continue taking part in the subsequent questionnaires and whether they prefer paper or digital contact. After the second questionnaire (‘Q2’) onwards, only participants who specifically agreed to receive the third and subsequent questionnaires were sent follow-up questionnaires. Data were digitally entered by research staff in Castor EDC, Amsterdam, the Netherlands. Individuals who opted for digital participation were provided a link by email to directly enter their responses in Castor EDC. Returned questionnaire data were checked for quality and consistency, and subsequently coded by a team of experienced researchers to prepare for data analysis.

### Case definition COVID-19

Participants were classified as ‘definite’ COVID-19 cases if they reported a physician-confirmed diagnosis of COVID-19 with self-reported subsequent verification through PCR testing. Participants were classified as ‘probable’ COVID-19 cases if they reported a physician-confirmed diagnoses without subsequent verification through PCR testing or with missing data on the latter. Participants were classified as ‘possible’ COVID-19 cases if they stated to have experienced a COVID-19 infection either accompanied by a physician-confirmed diagnosis or not. Finally, participants were classified as non-cases if all previous classifications were not applicable or if there were missing answers to components of the abovementioned algorithms. For analyses presented in this paper, definite and probable cases were classified as a COVID-19 case. All other participants were defined as a non-case.

### Other covariates

The core protocol of the Rotterdam Study includes home interviews of all participants, repeated every 3–6 years. Since such regular activities of the Rotterdam Study were suspended soon after the first emergence of COVID-19 in the Netherlands in February 2020, we leveraged the most recent home interview to obtain information on demographics, pre-lockdown lifestyle and medical history. Marital status was categorized as living with or without partner. Educational attainment was categorised as primary education (‘primary’), lower/intermediate general education or lower vocational education (‘lower’), intermediate vocational education or higher general education (‘further’), or higher vocational education or university (‘higher’).

### Statistical analysis

Interrater reliability was assessed in a random subset of 10% of questionnaires using the Cohen’s Kappa Coefficients. Data from the Dutch National Institute for Public Health and the Environment on the nation-wide time-specific prevalence of COVID-19 infection in the Netherlands was obtained and visualized against calendar time of sending out the COVID-19 questionnaire (https://www.rivm.nl/coronavirus-covid-19/actueel). The severity of governmental countermeasures was summarized using the validated COVID-19 Government stringency index [7]. This is a composite measure based on nine response indicators, including school and workplace closures, restrictions on public gatherings, travel bans and stay-at-home requirements. It is scaled to a value from 0 to 100 (100 = strictest). This study is reported according to the Strengthening the Reporting of Observational Studies in Epidemiology (STROBE) guidelines. All analyses were performed at the significance level of 0.05 (2-tailed) using SPSS Statistics version 24.0.0.1 (IBM, Armonk, NY) and R version 3.4.3 (R Foundation for Statistical Computing, Vienna, Austria, www.R-project.org).

## Results

### Response rates

In Table 1 the response rates across the six questionnaires are shown. Initially, all 8732 participants within the Rotterdam Study that were alive and non-institutionalized on April 20th 2020, were sent the first questionnaire. Given the rapidly changing circumstances of the emerging pandemic and societal response at that time, we sent out the second questionnaire to the entire cohort after 3 weeks, on May 7th 2020, without awaiting actual response and willingness to participate from the first questionnaire. Based on participants’ responses on Q1 and Q2, we identified 5613 participants that were willing to participate in subsequent questionnaires from Q3 onwards. For these follow-up questionnaires, participants were provided an option to either fill out the questionnaire on paper or digitally. Approximately 2500 (43.3%) of 5613 participants agreed to receive the questionnaire digitally. The response to the digital questionnaires was slightly higher (81.6%) as compared to those returned on paper (73.5%). In total, 30,726 questionnaires were returned across the six waves of questionnaire rounds with an overall response rate of 75.5%.

**Table 1:** Number of sent and returned questionnaires across rounds as of April 28, 2021

	Date sent	Paper		Digital		Total	
		Number sent	Number returned	Number sent	Number returned	Number sent	Number returned
Questionnaire 1	20th April, 2020	8732	6241*	NA	NA	8732	6241*
Questionnaire 2	7th May, 2020	8687	5650	NA	NA	8687	5650
Questionnaire 3	22th May, 2020	3182	2824	2426	2053	5608	4877
Questionnaire 4	24st June, 2020	3493	2741	2470	2038	5963	4779
Questionnaire 5	30th July, 2020	3354	2456	2508	1997	5862	4453
Questionnaire 6	15th October, 2020	3322	2711	2495	2002	5817	4713
Total		30,770	22,623	9899	8090	40,669	30,713

*Response for questionnaire 1 is shown at time of questionnaire 1 data freeze for analysis, that is August 28, 2020. From August 28, 2020 up until April 28, 2021, five additional Q1 questionnaires have been returned

### Collection of data in relation to SARS-CoV-2 infection rates and governmental countermeasures

**Fig. 2:**
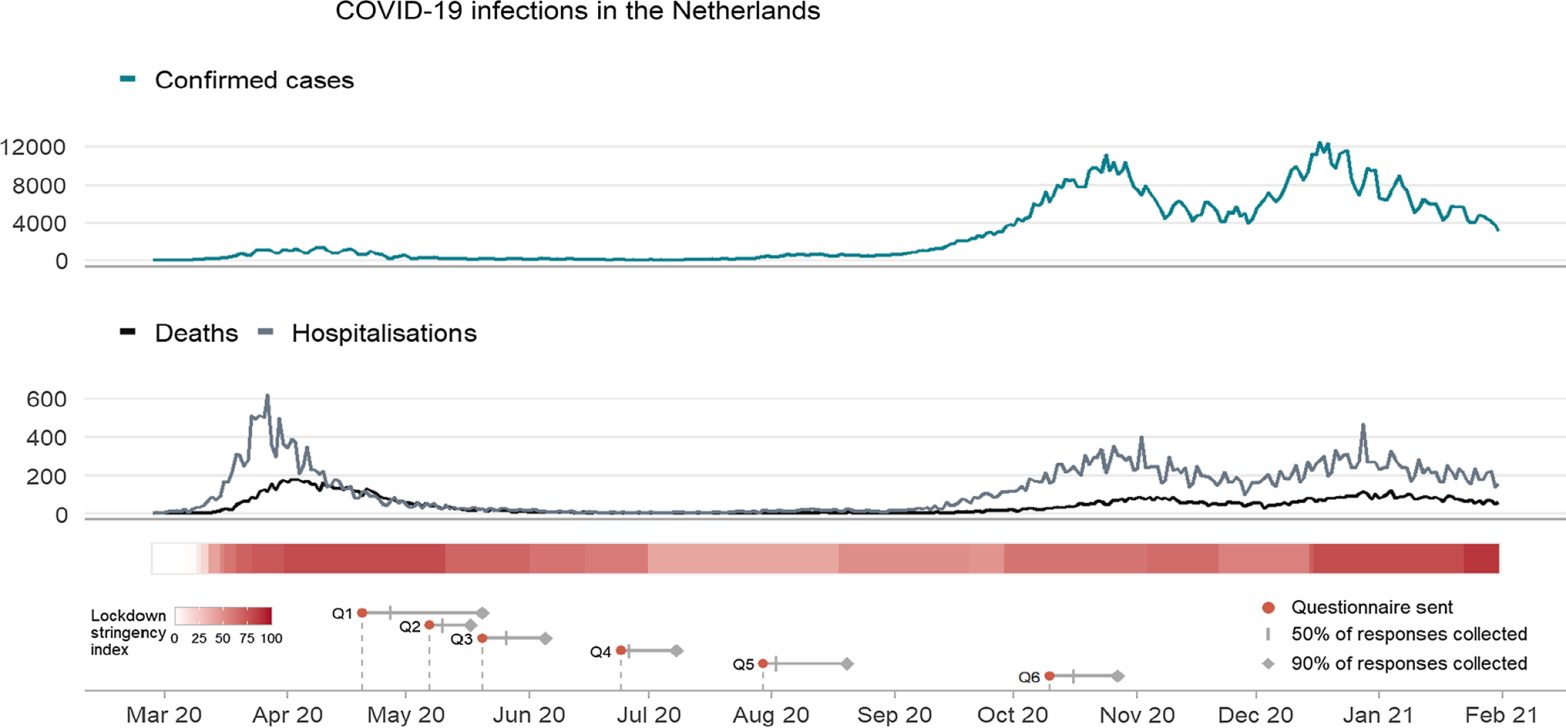
Timeline showing the dates of sending out the questionnaires in relation to the national numbers of newly confirmed COVID-19 cases, and COVID-19 related hospitalizations and mortality in the Netherlands

Figure 2 presents the temporal relation of data collection from the six questionnaires aligned with developments in SARS-CoV-2 infections, hospitalizations and mortality rates in the Netherlands (based on 93.1% of available data on exact date of return). In parallel, the figure summarizes the severity of Dutch governmental countermeasures in terms of the stringency index in relation to the collection of data. A detailed overview of all taken countermeasures across calendar time is presented in the Supplementary Information, eFig. 2.

### Baseline demographics

To report on baseline demographics of the overall study population, we used data from the first COVID-19 questionnaire (Q1). Table 2 presents the baseline demographics of the participants that returned the first COVID-19 questionnaire (71.5%), with an average reliability coefficient of 95% as assessed by Cohen’s Kappa. Mean age at baseline was 70.2 years (range 44–102 years), and women represented 58.4% of the population. Non responders were more often women (61%), and slightly younger (mean 68.9 years) than those that responded to the questionnaire (eTable 1). The majority of participants indicated to be in a good health condition (57.%) by self-report, whereas only 1.2% indicated that they found themselves to be in a poor health condition. Approximately two-thirds of all participants reported to have a history of any chronic disease (65.8%). Between April 20th and July 10th 2020, 329 (5.3%) of all participants reported to have had a COVID-19 infection, of whom 14 were defined as definite, 58 as probable and 257 as possible COVID-19 cases. Individuals who reported to be infected with COVID-19 were generally younger than their uninfected peers, and more often had a history of chronic lung disease or mental health illnesses (eTable 2).

**Table 2:** Characteristics of the study population that responded to the first questionnaire (*N* = 6241)

Characteristics	
Age (mean [SD])	70.16 (11.63)
Women	3643 (58.4)
Education	
Primary	409 (6.6)
Lower/intermediate or lower vocational	2095 (33.6)
Intermediate vocational or higher general	1977 (31.7)
Higher vocational or university	1698 (27.2)
Self-appreciation of health	
Excellent	432 (7.5)
Very good	1195 (20.6)
Good	3340 (57.6)
Fair	761 (13.1)
Poor	69 (1.2)
Self-report of chronic diseases	3815 (61.1)*
Cancer	852 (13.7)
Cardiovascular disease	1702 (27.3)
Stroke	445 (7.1)
Chronic lung disease	827 (13.3)
Neurodegenerative disease	99 (1.6)
Diabetes	570 (9.1)
Mental illness	266 (4.3)
Other^#^	1159 (18.6)

Data are presented as N (% of the total study population), unless stated otherwise. *Counts of individual diseases exceed 61.1%, since participants could report more than one disease. SD: standard deviation; Q1: the first questionnaire. ^#^Other self-reported diseases primarily included osteoarthritis (*N* = 149, 2.4%), hay fever (*N* = 67, 1.1%) and asthma (*N* = 22, 0.4%). Less than 7.1% of data were missing: education (*N* = 52), self-appreciation of health (*N* = 444) and self-report of chronic diseases (*N* = 442)

## Discussion

This report showcases the design, implementation and initial findings of a dedicated COVID-19 questionnaire nested within a prospective, population-based cohort study; the Rotterdam Study. Although numerous reports have been published on the prevalence and risk factors of COVID-19 in the general population, very little data comes from studies that are nested within ongoing cohorts [8, 9]. Nesting (repeated) questionnaires within an ongoing cohort study has several major advantages over these ‘stand-alone’ questionnaires or registry studies. First, embedding a questionnaire in a closed population-based cohort study facilitates assessment of a wide range of systematically collected determinants that predispose to SARS-CoV-2 contraction or transmission in the general population, such as genetic or lifestyle factors [9]. Second, it allows for the determination of long-term consequences on general health and wellbeing of COVID-19 cases itself, yet also provides an opportunity to quantify collateral damage of governmental countermeasures to mental health or the consequences of healthcare avoidance during a pandemic.

In this population-based study, self-reported prevalence of COVID-19 was approximately 1%. Compared to national averages at the time, this relatively low point-prevalence should be interpreted with caution, as it is likely to be underestimated due to the following reasons. Back in April–May 2020, test capacity in the Netherlands was limited and identification of cases primarily relied on self-reported clinical symptoms instead of case-confirmed PCR and/or serum samples. It is also likely that participants that suffered more severe COVID-19 were not able to return a questionnaire due to debilitating sickness or hospital admission.

This study has several strengths and limitations. A major strength includes the design of a questionnaire within an existing cohort study. Robust infrastructure facilitated swift and flexible data collection, providing both paper as well as digital options to ensure limited selection bias, while a rapid collection of population-level data limited recall bias (80% of all questionnaires was returned in approximately 7 weeks after the first official case of COVID-19 in the Netherlands was confirmed). The longitudinal design of the study facilitates for long-term investigation of the consequences of the actual infection as well as accompanying countermeasures on several systematically collected health outcomes. Importantly, these health outcomes have been systematically collected within this study over several decades, allowing comparisons of potential differences in disease incidence before and after the pandemic. Several limitations have to be acknowledged. First, although overall response on the questionnaires was high (76%), response tended to decline in subsequent questionnaires. Second, by design, questionnaire data are self-reported and may differ when verifying those with data retrieved from medical records. Third, the vast majority of participants is of Caucasian descent (92%), limiting generalizability of findings to other ethnicities. Finally, questionnaires were sent at irregular time intervals to match with the actual SARS-CoV-2 infection rate and subsequently returned at varying pace, warranting potential adjustments for calendar time when comparing results across questionnaires. Nevertheless, the majority of questionnaires was returned within 4 weeks.

## Conclusions

This article described the design, considerations, implementation and initial results of the COVID-19 questionnaire within the Rotterdam Study. Ongoing on work is focused on mental health, avoidance of care and long-term consequences of COVID-19. It also showcases the importance of population-based cohort studies during a pandemic by demonstrating how existing infrastructure can be readily leveraged for population-based investigations on emerging diseases, in this case COVID-19.

## Supplementary Information

Below is the link to the electronic supplementary material.

Supplementary file1 (DOCX 177 kb)

## Data Availability

Data can be obtained upon request. Requests should be directed towards the management team of the Rotterdam Study (secretariat.epi@erasmusmc.nl), which has a protocol for approving data requests. Because of restrictions based on privacy regulations and informed consent of the participants, data cannot be made freely available in a public repository.
