# Supplementary material for: Design, implementation and initial findings of COVID-19 research in the Rotterdam Study: leveraging existing infrastructure for population-based investigations on an emerging disease
Source: Eur J Epidemiol. 2021 Jul 17;36(6):649–54. doi: 10.1007/s10654-021-00789-7 (PMC8286166; doi:10.1007/s10654-021-00789-7)
Supplement: Supplementary file 1 — Supplementary file1 (DOCX 177 kb) [file 10654_2021_789_MOESM1_ESM.docx]

**Design, implementation, and initial findings of COVID-19 research in the Rotterdam Study: leveraging existing infrastructure for population-based investigations on an emerging disease**

S. Licher^1^, N. Terzikhan^1^, M.J. Splinter^1^, P. Velek^1^, F. J.A. van Rooij^1^, J. Verkroost-van Heemst^1^, A.E.G. Haarman^1,2^, E.F. Thee^1,2^, S. Geurts^1^, M.M.J. Mens^1^, N. van der Schaft^1^, M. de Feijter^1^, L.M. Pardo^3^, B.C.T. Kieboom^1,4^, M.A. Ikram^1^

^1^ Department of Epidemiology, Erasmus MC - University Medical Center Rotterdam, Rotterdam, the Netherlands

^2^ Department of Ophthalmology, Erasmus MC - University Medical Center Rotterdam, Rotterdam, the Netherlands

^3^ Department of Dermatology, Erasmus MC - University Medical Center Rotterdam, Rotterdam, the Netherlands

^4.^ Department of General Practice, Erasmus MC - University Medical Center Rotterdam, Rotterdam, the Netherlands

Corresponding Author: M. Arfan Ikram, MD, PhD, Department of Epidemiology, Erasmus MC - University Medical Center Rotterdam, Rotterdam, the Netherlands; Telephone +31 10 704 34 88; Fax +31 10 704 46 57;

E-mail [m.a.ikram@erasmusmc.nl](mailto:m.a.ikram@erasmusmc.nl)

Supplementary methods

Detailed descriptions of questionnaire domains

General demographics: including age, sex and weight.

Household: household information, including housemate members, including children and their stress levels (due to the pandemic).

Pets: when applicable which pets participants had and how many.

Employment: current work situation, i.e. ‘What do you do in everyday life?’ with corresponding response categories ‘I work (fulltime, part-time, self-employed)’, ‘I’m on sick leave’, ‘unemployed’, ‘retired’, or ‘other’.

Health status: participants’ self-appreciation about their own health, lifestyle, physical activity, current and new diseases or symptoms, and medication use. Participants were asked how they perceived their own health in general. This was also done for a list of specific symptoms that were considered relevant; under which headache, chest pain, dyspnoea and loss of smell and taste.

Medical history: self-reported number and type of non-communicable diseases

COVID-19 infection and impact: whether participants were positively tested for covid-19, or assumed they underwent a covid-19 infection, whether they have been in close contact with an infected person, whether they have been hospitalized, got oxygen treatment, entered the ICU or have been intubated. To assess corona impact on participants daily life, participants were asked to rate their quality of life, to state whether they trust the Dutch government, whether they concerns regarding the pandemic and its impact on their health, the health of family or friends and about their job security.

Medication use: Continuous linkage with dispensing records from pharmacies in the study area with the study database provides daily information on drug use for all study participants. These records contain automated information on all dispensed prescriptions and include product names, international non-proprietary names, Anatomical Therapeutic Chemical (ATC) codes, number of filled tablets/capsules or other dosage forms, dates of delivery, prescribed daily numbers, dosages and legend durations (prescription length).

Mental health: Respondents were screened for depressive symptoms using ten out of twenty questions from the Center for Epidemiological Studies Depression (CESD) scale, with a weighted maximum score of 29. The higher the score on this scale, the more depressive symptoms participants experienced during the week before completing the questionnaire. Anxiety was measured by seven of out fourteen questions from the Hospital Anxiety and Depression Scale (HADS), which has a weighted maximum score of 20.

Vaccines: In Q1 participants were asked whether they were vaccinated according to the Dutch National vaccination program or were administered the flu vaccine in the preceding year. In Q6 participants were also asked whether they would consider taking the COVID-19 vaccine.

Social life and relations: including isolation or loneliness, and the level of connection to Dutch inhabitants, neighbours, family and/or friends.

Lifestyle: changes in lifestyle during the pandemic (physical activity, diet, smoking and alcohol use).

Travel: travel information, including whether participants went abroad just before the emergence of SARS-CoV-2 pandemic in the Netherlands.

New skin diseases: presence of dermatological diseases that potentially could be related to COVID-19, including for example erythema multiforma or urticaria.

Healthcare utilization: Participants were asked whether they had experienced symptoms for which they otherwise would have contacted their general practitioner or medical specialist, but now did not do so because of COVID-19. They were provided with a pre-specified list of both symptoms that might have warranted urgent medical assessment and generic symptoms, which made it possible for participants to indicate for which symptoms they had avoided healthcare: palpitations, chest pain, limb weakness, self-perceived cancer-related symptoms (e.g. weight loss, suspicious skin spots), difficulty speaking or facial drooping, vision loss, elevated blood pressure, sudden onset dizziness, dysregulation of diabetes, nausea, fluid retention (oedema), memory complaints, attempts to stop or reduce smoking, and lower back pain. Additionally, we inquired participants whether healthcare appointments or check-ups have been cancelled or postponed due to COVID-19.

Contact/follow-up: Participants were asked to give consent to receive follow-up questionnaires every two weeks, and whether they would rather prefer to receive a digital questionnaire by email instead of a paper questionnaire by mail.

eTable 1. Characteristics of the study population at sending of first questionnaire (N=8,732)

|  | **Total invited for questionnaires** | **Non-responder** | **Responder** | **P-value** |
| --- | --- | --- | --- | --- |
| Number | 8,732 | 2,491 | 6,241 |  |
| Age at receipt of first questionnaire (SD) | 69.8 (12.4) | 68.9 (14.0) | 70.2 (11.6) | **<0.01** |
| Women (%) | 5,162 (59) | 1,519 (61) | 3,643 (58) | **0.03** |
| Beyond primary education (%) | 2,212 (26) | 514 (21) | 1,698 (27) | **<0.01** |
| Caucasian ethnicity (%) | 7,794 (92) | 2,085 (87) | 5,709 (94) | **<0.01** |

Data are presented as numbers (%) or mean ± standard deviation (SD). Bold P-values represent statistical significance. Ethnicity was based on questionnaire data and was categorized according to the country of birth of both parents and grandparents of the participants

# eTable 2. Detailed characteristics of the study population based on first questionnaire responses, stratified by COVID-19 status

|  |  | **COVID-19 classification** | |  | **P-value for difference across the levels of COVID-19 classifications** |
| --- | --- | --- | --- | --- | --- |
|  | **Non-cases** | **Definite** | **Probable** | **Possible** |  |
| **N** | 5,516 | 14 | 58 | 257 |  |
| Age (mean (SD)) | 70.4 (11.4) | 62.9 (11.3) | 62.88 (11.0) | 62.88 (11.0) | **<0.001** |
| Women (%) | 3,204 (58.1) | 9 (64.3) | 42 (72.4) | 145 (56.4) | 0.145 |
| Education |  |  |  |  | **<0.001** |
| Primary | 343 (6.2) | 0 | 3 (5.2) | 11 (4.3) |  |
| Lower/intermediate or lower vocational | 1858 (33.7) | 3 (21.4) | 17 (29.3) | 63 (24.5) |  |
| Intermediate vocational or higher general | 1770 (32.1) | 5 (35.7) | 21 (36.2) | 83 (32.3) |  |
| Higher vocational or university | 1492 (27.0) | 6 (42.9) | 17 (29.3) | 97 (37.7) |  |
| **Self-appreciation of health (%)** |  |  |  |  | **0.033** |
| Excellent | 403 (7.4) | 0 (0.0) | 2 (3.5) | 25 (9.8) |  |
| Very good | 1,124 (20.6) | 2 (14.3) | 9 (15.8) | 55 (21.5) |  |
| Good | 3,155 (57.9) | 10 (71.4) | 32 (56.1) | 126 (49.2) |  |
| Fair | 696 (12.8) | 2 (14.3) | 14 (24.6) | 48 (18.8) |  |
| Poor | 67 (1.2) | 0 (0.0) | 0 (0.0) | 2 (0.8) |  |
| **Self-report of chronic disease (%)** | 3,580 (65.8) | 10 (71.4) | 47 (81.0) | 157 (61.3) | **0.038** |
| Cancer (%) | 811 (20.7) | 1 ( 9.1) | 8 (15.7) | 29 (18.4) | 0.549 |
| Cardiovascular disease (%) | 1,615 (46.4) | 3 (27.3) | 15 (34.1) | 64 (43.5) | 0.198 |
| Chronic lung disease (%) | 754 (13.7) | 4 (28.6) | 20 (34.5) | 45 (17.5) | **<.001** |
| Neurodegenerative disease (%) | 95 (1.7) | 0 | 0 | 4 (1.6) | **0.666** |
| Diabetes Mellitus (%) | 536 (9.7) | 3 (21.4) | 5 (8.6) | 22 (8.6) | **0.526** |
| Mental illness (%) | 234 (4.2) | 0 | 9 (15.5) | 22 (8.6) | **<0.001** |
| Other (%) * | 1,083 (19.6) | 2 (14.3) | 15 (25.9) | 57 (22.2) |  |

Data are presented as n (%) or Median ± SD. Bold P-values represent statistical significance. SD: standard deviation; TNA: transient ischemic attack. *Other self-reported diseases primarily included osteoarthritis (N=149, 2.4%), hay fever (N=67, 1.1%), and asthma (N=22, 0.4%).

**N=18,924** Total number of participating individuals in the Rotterdam Study

**N=10,192** Number of participants that died, were institutionalized or did not provide informed consent for follow-up on or before April 8^th^, 20202020

**N=8,732** (100%) Number of living and actively participating individuals on April 8^th^, 2020

**N=2,491** (28.5%) Number of non-responders

**N=6,241** (71.5%) Number of responders on Q1

**eFigure 1**. Flow chart of participants in the study.


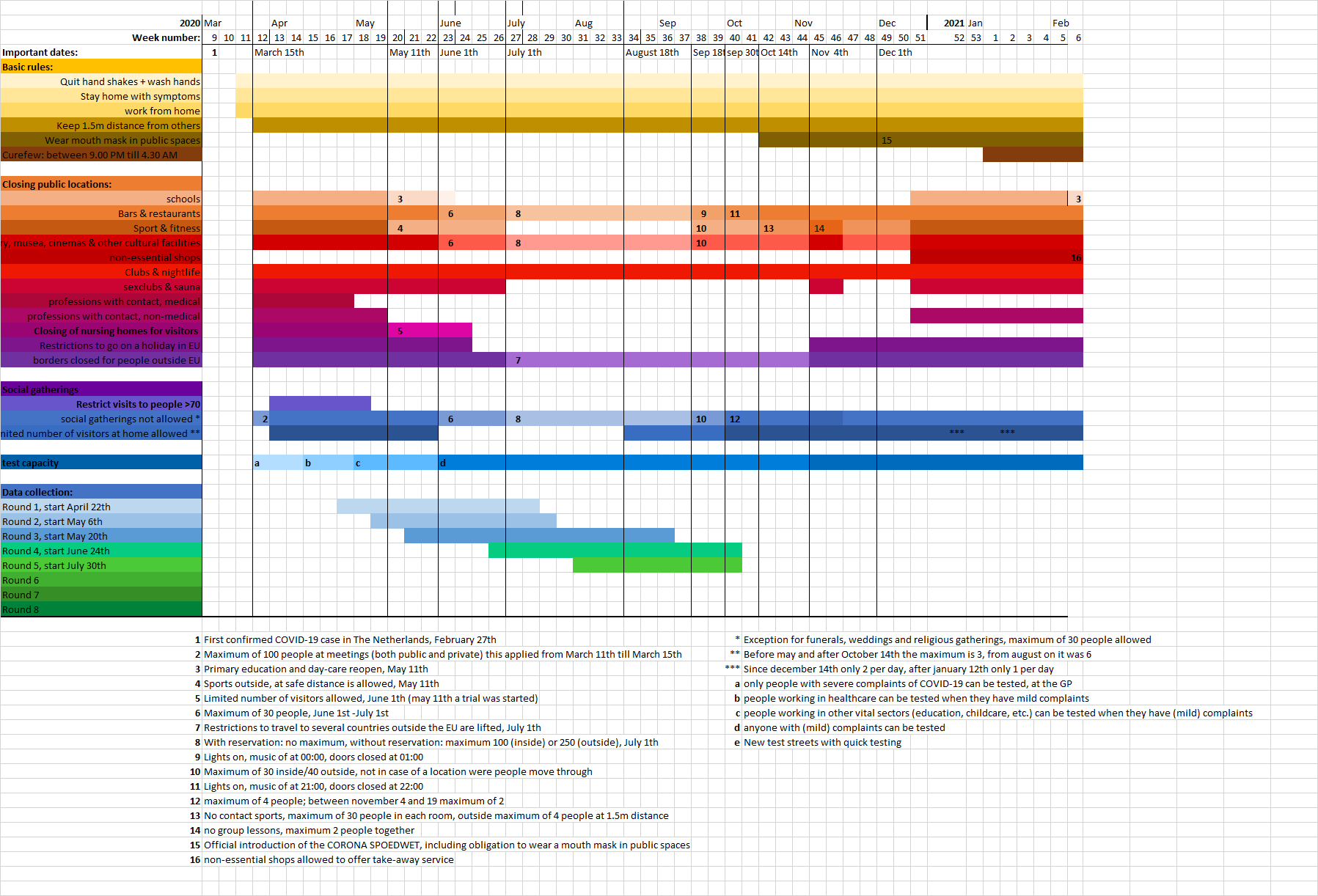
**eFigure 2**. Detailed timeline of governmental countermeasures in the Netherlands
